# Elucidation of Cross-Talk and Specificity of Early Response Mechanisms to Salt and PEG-Simulated Drought Stresses in *Brassica napus* Using Comparative Proteomic Analysis

**DOI:** 10.1371/journal.pone.0138974

**Published:** 2015-10-08

**Authors:** Junling Luo, Shaohua Tang, Xiaojue Peng, Xiaohong Yan, Xinhua Zeng, Jun Li, Xiaofei Li, Gang Wu

**Affiliations:** 1 Key Laboratory of Biology and Genetic Improvement of Oil Crops, Ministry of Agriculture of People’s Republic of China, Oil Crops Research Institute, Chinese Academy of Agricultural Sciences, Wuhan, China; 2 Key Laboratory of Molecular Biology and Gene Engineering of Jiangxi Province, College of Life Science, Nanchang University, Nanchang, China; Wuhan University, CHINA

## Abstract

To understand the cross-talk and specificity of the early responses of plants to salt and drought, we performed physiological and proteome analyses of *Brassica napus* seedlings pretreated with 245 mM NaCl or 25% polyethylene glycol (PEG) 6000 under identical osmotic pressure (-1.0 MPa). Significant decreases in water content and photosynthetic rate and excessive accumulation of compatible osmolytes and oxidative damage were observed in response to both stresses. Unexpectedly, the drought response was more severe than the salt response. We further identified 45 common differentially expressed proteins (DEPs), 143 salt-specific DEPs and 160 drought-specific DEPs by isobaric tags for relative and absolute quantitation (iTRAQ) analysis. The proteome quantitative data were then confirmed by multiple reaction monitoring (MRM). The differences in the proteomic profiles between drought-treated and salt-treated seedlings exceeded the similarities in the early stress responses. Signal perception and transduction, transport and membrane trafficking, and photosynthesis-related proteins were enriched as part of the molecular cross-talk and specificity mechanism in the early responses to the two abiotic stresses. The Ca^2+^ signaling, G protein-related signaling, 14-3-3 signaling pathway and phosphorylation cascades were the common signal transduction pathways shared by both salt and drought stress responses; however, the proteins with executive functions varied. These results indicate functional specialization of family proteins in response to different stresses, i.e., CDPK21, TPR, and CTR1 specific to phosphorylation cascades under early salt stress, whereas STN7 and BSL were specific to phosphorylation cascades under early drought stress. Only the calcium-binding EF-hand family protein and ZKT were clearly identified as signaling proteins that acted as cross-talk nodes for salt and drought signaling pathways. Our study provides new clues and insights for developing strategies to improve the tolerance of crops to complex, multiple environmental stresses.

## Introduction

*Brassica napus* is the third most economically important oilseed crop in the world and is a essential source of plant oil and proteins for human consumption and animal feed [1]. With global climate changes and industrialization, drought and salt have become major challenges to oilseed production [2]. Improving the tolerance of oilseed crops to drought and salt is an important strategy to increase oilseed yield and supply. Understanding the mechanisms of both drought and salt in oilseed crops is critical. Compared to *Arabidopsis*, much less is known about the mechanism underlying oilseed responses to salt and drought stresses although some progress has been made recently [2–4].

Both salt and drought severely affect crop growth and production [5, 6]. Drought limits plant growth by imposing osmotic stress, whereas salt disrupts plant growth by causing physiological drought and ion toxicity [7]. Elucidating the universal and specific response mechanisms of drought and salt will provide insights on how plants integrate multiple signal pathways that may cross-talk or diverge at various steps in the response pathway [8]. Cross-talk among Ca^2+^ and ABA-signaling pathways and mitogen-activated protein kinase (MAPK) cascades in drought and salt stress responses have recently been reported [8–10]. As a second messenger, calcium is a major factor in signaling cross-talk because it can be induced by both salt and drought. ABA is an important phytohormone that plays a critical role in response to various stress signals, particularly, in regulating plant water balance. MAPKs connect diverse receptors/sensors to a wide range of abiotic stresses, including drought, salt and cold. Members of MAPK cascades are activated in plants in response to various stresses, suggesting MAPKs act as cross-talk nodes in stress signaling [8–10]. Although conventional forward and reverse genetic approaches have provided valuable insights into stress responses, technical limitations may prevent further development. To compensate for the defects of traditional technologies, genome-wide transcriptome and proteome strategies have been applied. Using these-omics approaches, a large number of salt and drought stress-related genes or proteins have been identified [11, 12]. However, no systematic consensus on the specific categories of proteins that correspond to particular signaling events has been established so far. The complexity of signaling networks is far beyond our imagination. Identification of key factors at the nodes and branches between different stress-response pathways will aid in understanding the mechanism of stress responses in higher plants. Much effort is required to elucidate the critical points of convergence and divergence that enable the integration of different signals.

Proteomic technologies are an effective approach to conduct large-scale, quantitative, and reproducible analyses of the proteomic profiles of a stress responses. Moreover, mass spectrometry (MS)-based proteomic analysis is a powerful tool to identify isoforms of specific proteins, thus enabling the specific and overlapping functions within a protein family to be distinguished. Using this strategy, most of the previous proteomic analyses of abiotic stress responses in higher plants have focused on the responses to long-term (≥ 24 h) exposure to stress [13]. Only a few studies have focused on the early (≤ 24 h) transcriptome and physiological responses [14–17]. Very limited proteome data are available about the early events in the perception and transduction of drought and salt stress signals [16, 18]. Recent preliminary studies have demonstrated that the response of plants to stress maybe determined by the rapid perception of stress shock that occurs within hours [14, 16]. However, these investigations did not extensively examine the systematic signaling network. Understanding the specificity and cross-talk between different stress stimuli is essential and may provide clues and insights for developing strategies to improve the tolerance of crops to complex multiple environmental stresses worldwide.

In the present study, NaCl and PEG were used as salt and osmotic stress agents, respectively, to imitate environment salt and drought stresses. We investigated the proteomic profiles of the early response of *B*. *napus* to salt and drought stresses using iTRAQ-tandem mass spectrometry (MS/MS) technology. We observed enrichment of membrane trafficking and signal perception and transduction proteins, suggesting that these proteins were crucial for elucidating the molecular cross-talk and specificity mechanism in the early response to the two abiotic stresses. A calcium-binding EF-hand family protein and ZKT protein were key proteins mediating the cross-talk between salt and drought stress responses, whereas different potential receptors, small G proteins, 14-3-3 proteins, kinases and phosphatase guided stress-specific late responses. Our results provided valuable insights to guide future studies of the cross-talk between the salt and drought stress responses.

## Materials and Methods

### Plant growth conditions and stress treatments

Seeds of a winter-type *B*. *napus* line, Zhong Shuang11 (ZS11), were germinated in Petri dishes in the dark at 23°C for 4 days. Seedlings were transferred to plastic plots filled with half-strength (1/2) Hoagland’s nutrient medium and grown in a growth chamber in a controlled environment (16 h photoperiod, 350 μmol m^−2^ s^−1^, 22 ± 2°C, and 65%–70% relative humidity).

The osmotic pressures of different concentrations of NaCl solution (200 mM, 215 mM, 230 mM, 245 mM, 250 mM, and 260 mM) and PEG 6000 solution (18%, 20%, 22%, 24%, 25%, and 26%) (w/v) were detected using a freezing-point depression osmometer (Osmomat 030, Gonotec, Berlin, Germany). Isotonic solutions of 245 mM NaCl and 25% PEG 6000 (which showed an identical osmotic potential of -1.0 MPa) were selected for further stress-treatment experiments.

For proteome analysis and physiological index determination, 15-day-old seedlings were randomly assigned to the control and stress treatments. After treatment for 4 h, true leaves were harvested for further experiments. For proteomic analysis, 60 seedlings in one plastic pot were used for each treatment; six independent experiments were performed for each treatment, and two biological repeats were pooled from the leaf samples of three experiments per treatment. For physiological analysis, three biological and technical repeats were performed for each treatment.

### Physiological analysis

The relative water content was measured according to with the methodology of Pang *et al*. (2010) [11]. Total chlorophyll was isolated using an extraction solution (2:1 acetone:95% alcohol), and the chlorophyll *a* and *b* contents were measured at 646.6, 663.6, and 750 nm using a spectrophotometer (Lambda, PerkinElmer, USA), as described elsewhere [19]. The photosynthetic and transpiration rates of leaves were assessed using a LI-6400XT portable photosynthesis system (LI-COR Biosciences, Lincoln, NE, USA). Chlorophyll fluorescence was measured using an Imaging PAM 101 (Walz, Effeltrich, Germany), and F_v_/F_m_ values were calculated according to the manufacturer’s instructions. Proline concentrations were estimated using the ninhydrin reaction method [20, 21]. The concentrations of total soluble sugars and sucrose were determined as previously described [21]. Malondialdehyde (MDA) was extracted and measured as described elsewhere [22]. For the H_2_O_2_ content assay, plant leaves were homogenized in sodium phosphate buffer, and the supernatant and 0.1% (w/v) titanium sulfate reagent were mixed to precipitate the peroxide-titanium complex. The absorbance of the solution was quantified at a wavelength of 410 nm.

### Protein extraction and digestion

Approximately 1 g of fresh leaves was frozen and ground to a fine powder in liquid nitrogen. Total protein was extracted in lysis buffer [7 M urea, 2 M thiourea, 4% CHAPS, 40 mM Tris-HCl (pH 8.5), 1 mM PMSF, and 2 mM EDTA] and then sonicated for 15 min. A 5× volume of chilled acetone was added to the protein mixtures, and the proteins were precipitated at -20°C for 2 h. The pellets obtained after centrifugation at 25,000×*g* for 20 min were resuspended in lysis buffer. The protein extracts were reduced with 10 mM DTT (final concentration) at 56°C for 1 h, followed by precipitation with a 5× volume of chilled acetone at -20°C for 2 h. After centrifugation at 25,000×*g* at 4°C, the pellets were dissolved in 0.5 M TEAB (Applied Biosystems, Milan, Italy) and sonicated on ice. After centrifugation at 25,000×*g* at 4°C, an aliquot of each supernatant was used to determine the protein concentration using Bradford assay reagents and a spectrophotometer (UV-160, Shimadzu, Kyoto, Japan). Bovine serum albumin was used as the protein standard. The supernatant proteins were stored at -80°C until proteomic analysis.

For digestion, total protein (100 μg) was digested with Trypsin Gold (Promega, Madison, WI, USA) at a 30:1 protein:trypsin ratio at 37°C for 16 h.

### iTRAQ labeling and strong cation exchange (SCX) fractionation

After trypsin digestion, peptides were dried by vacuum centrifugation. Peptides were reconstituted in 0.5 M TEAB and labeled using an 8PLEX iTRAQ^®^ Reagent Kit (AB Sciex Inc., Foster City, CA, USA) following the manufacturer’s recommendations. The untreated controls were labeled with iTRAQ tags 113 (biological repeat 1) and 117 (biological repeat 2), the salt-treated samples were labeled with iTRAQ tags 114 (biological repeat 1) and 118 (biological repeat 2), and the drought-treated samples were labeled with iTRAQ tags 115 (biological repeat 1) and 119 (biological repeat 2). The labeled peptides were incubated at room temperature for 2 h, then pooled and vacuum-centrifuged to dryness.

SCX chromatography was performed with a LC-20AB HPLC pump system (Shimadzu, Kyoto, Japan). The iTRAQ-labeled peptide mixtures were reconstituted in 4 mL of buffer A (25 mM NaH_2_PO_4_ in 25% ACN, pH 2.7) and loaded onto a 4.6 × 250 mm Ultremex SCX column containing 5-μm particles (Phenomenex). The peptides were eluted at a flow rate of 1 mL/min using a gradient of buffer A for 10 min, 5%–60% buffer B (25 mM NaH_2_PO_4_, 1 M KCl in 25% ACN, pH 2.7) for 20 min, and 60%–100% buffer B for 2 min. The system was then maintained at 100% buffer B for 1 min before equilibrating with buffer A for 10 min prior to the next injection. Elution was monitored by measuring the absorbance of the eluent at a wavelength of 214 nm, and fractions were collected at 1-min intervals. The eluted peptides were pooled into 20 fractions, desalted with a Strata X C18 column (Phenomenex) and vacuum-dried.

### Liquid chromatography—electrospray ionization—tandem mass spectrometry

Each fraction was resuspended in buffer A (5% ACN, 0.1% FA) and centrifuged at 20,000×*g* for 10 min. The average final peptide concentration was approximately 0.5 μg/μL. Five micrograms of peptide was loaded onto a LC-20AD nanoHPLC (Shimadzu, Kyoto, Japan) using an autosampler and a 2-cm C18 trap column. The peptide was then eluted from a 10-cm analytical C18 column (inner diameter: 75 μm) packed in-house. The samples were loaded at 8 μL/min for 4 min, then a 35-min gradient was run at 300 nL/min starting from 2% to 35% B (95% ACN, 0.1% FA), followed by a 5-min linear gradient to 60%, followed by a 2- min linear gradient to 80%, maintained at 80% for 4 min, and finally returned to 5% in 1 min.

Data processing was performed using a TripleTOF 5600 System (AB SCIEX, Concord, ON, Canada) fitted with a NanoSpray III source (AB SCIEX, Concord, ON, Canada) and a pulled-quartz tip as the emitter (New Objectives, Woburn, MA, USA). For information-dependent acquisition, survey scans were acquired at 250 ms, and as many as 30 product ion scans exceeding a threshold of 120 counts/s and with a 2+ to 5+ charge-state were collected. The total cycle time was fixed at 3.3 s. The Q2 transmission window was 100 Da for 100%. Four time bins were summed for each scan at a pulser frequency of 11 kHz by monitoring the 40-GHz multichannel TDC detector with four-anode channel detection. A sweeping collision energy setting of 35±5 eV coupled with iTRAQ-adjusted rolling collision energy was applied to all precursor ions for collision-induced dissociation. Dynamic exclusion was set for half the peak width (15 s), and the precursor was then removed from the exclusion list.

### Proteomic data analysis

The raw data files acquired from the Orbitrap were converted to MGF files using Proteome Discoverer 1.2 (Thermo Fisher Scientific, Bremen, Germany). Protein identification was performed using the Mascot search engine (version 2.3.02; Matrix Science, London, UK) against two *B*. *napus* genome databases of an unpublished database containing 83,006 sequences and a published database containing 98,762 sequences (http://www.genoscope.cns.fr/brassicanapus/data/). Mass tolerance of 0.05 Da (ppm) was permitted for intact peptide masses and 0.1 Da for fragmented ions, with one missed cleavage allowed in the trypsin digests. Gln->pyro-Glu (N-term Q), oxidation (M), and deamidated (NQ) were selected as the potential variable modifications, and carbamidomethyl (C), iTRAQ8plex (N-term), and iTRAQ8plex (K) were selected as fixed modifications. Specifically, an automatic decoy database search was performed in Mascot by choosing the decoy checkbox in which a random sequence from the database was generated and tested for the raw spectra and the true database. To reduce the probability of false peptide identification, only peptides with a significance score ≥20 at the 99% confidence interval from a Mascot probability analysis greater than “identity” were counted.

The minimum requirements for differentially expressed proteins (DEPs) were at least two matched unique peptides and a significant change (*P* < 0.05 and > or < 1.2-fold) in protein quantities between the stress-treated samples and control samples in at least one replicate, with the other replicate displaying a similar trend. Functional annotation of DEPs was conducted using the Blast2GO program against NCBInr and Swiss-Prot databases. The Kyoto Encyclopedia of Genes, Genomes database (KEGG; http://www.genome.jp/kegg/) and the Clusters of Orthologous Groups of Proteins database (COG; http://www.ncbi.nlm.nih.gov/COG/) were used to classify the proteins.

### Protein validation by multiple reaction monitoring (MRM)

MRM was used to validate the DEPs. MRM transitions for selected proteins of interest were predicted using Skyline based on a protein FASTA database derived from a MS/MS spectra library imported from ProteinPilot software (AB SCIEX, Foster City, CA, USA) [23]. Samples were digested as described elsewhere [24] and spiked with 20 fmol of α-galactosidase for data normalization. MRM analyses were performed on a QTRAP 5500 mass spectrometer (AB Sciex Inc., Foster City, CA, USA) equipped with a Waters nanoACQUITY ultra-performance liquid chromatography system (Waters Corp., Milford, MA). The mobile phase consisted of 0.1% aqueous formic acid (solvent A) and 98% acetonitrile with 0.1% formic acid (solvent B). Peptides were separated on a BEH130 C18 column (0.075 × 200 mm column, 1.7 μm; Waters) at 300 nL/min, and eluted with a gradient of 5%−8% solvent B for 2 min, 8%−30% solvent B for 94 min, and 30%−80% solvent B for 3 min. For QTRAP 5500 mass spectrometry, a spray voltage of 2100 V, nebulizer gas of 20 p.s.i., and dwell time of 10 ms were applied. Multiple MRM transitions were monitored using a unit resolution in both Q1 and Q3 quadrupoles to maximize specificity. Data analysis was conducted using Skyline [23]. The three most abundant transitions for each peptide were used for quantitation unless interference from the matrix was observed.

### Statistical analysis

Student’s *t*-test was used for significance evaluation when only two groups were compared. The statistical significance of multiple comparison among groups was evaluated by one-way ANOVA. All calculations were performed using with software SPSS 19.0 (SPSS, Inc., USA).

## Results

### Early physiological response to drought and salt stresses

**Fig 1:**
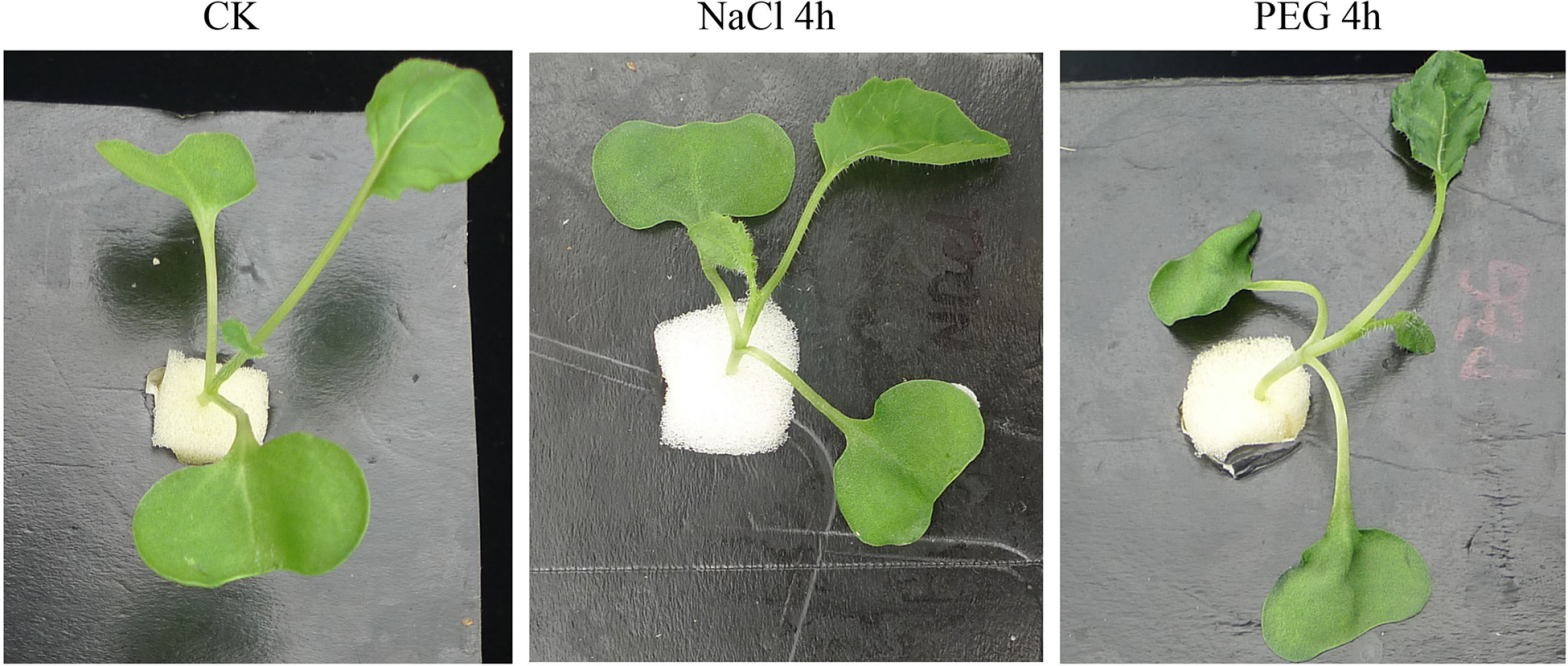
Phenotypic changes in *B*. *napus* seedlings exposed to 245 mM NaCl or 25% PEG 6000 for 4 h.

According to previous research, under the premise of iso-osmotic pressure, the major difference between salt and drought stress is ion toxicity caused by Na^+^ and Cl^-^ [7]. To eliminate differences due to different osmotic stressors, we determined the osmotic potential of different concentrations of NaCl and PEG 6000 solutions (S1 Table). The results indicated that 245 mM NaCl and 25% PEG 6000 exhibited identical osmotic potential (-1.0 MPa); therefore, 245 mM NaCl and 25% PEG 6000 were used in subsequent experiments. We hypothesized that the early responses of *B*. *napus* to both stresses would initially involve the promotion of primary signal perception and transduction, which could be more important than the later responses to stresses. We observed the stress responses of 15-day-old *B*. *napus* seedlings during the early stage of the treatments. After treatment for 4 h, we observed visible phenotypic changes in both stress treatments (Fig 1), namely, hypocotyl bending and cotyledon wilting. The stress symptoms under drought stress were more severe than those under salt stress. These physiological changes represented the plant’s early response to these specific stresses.

We first evaluated the net moisture content of the leaf, stem and root after 4 h of stress treatment. The net moisture content decreased under both salt and drought stress treatments. As shown in Table 1, the moisture content decreased by 6.9% under salt stress and by 11.3% under drought stress in the leaf, 4.4% under salt stress and 8.8% under drought stress in the root, and 2.7% under salt stress and 7.4% under drought stress in the stem. These results indicated that the effects of water loss decreased in the order leaf>root>stem and were more severe under drought stress than under salt stress.

**Table 1:** Physiological and chemical parameters of *B*. *napus* seedlings after treatment with 245 mM NaCl or 25% PEG 6000 for 4 h.

Index		Control	Salt	Drought
Relative water content (%)	Leaf	87.4 ± 2.7 (a)	81.4 ± 1.6 (b)	77.5 ± 1.4 (c)
	Stem	82.3 ± 3.2 (a)	80.1 ± 1.5 (ab)	76.2 ± 0.8 (b)
	Root	83.4 ± 1.9 (a)	79.7 ± 3.5 (b)	76. 1± 1.5 (c)
Proline (μmol g^-1^ DW)		0.764 ± 0.004 (a)	1.092 ± 0.058 (b)	1.099 ± 0.099 (b)
Soluble sugar (mg g^-1^DW)		40.276 ± 3.331 (a)	47.694 ± 2.260 (b)	52.907 ± 1.086 (c)
Sucrose (mg g^-1^DW)		22.646 ± 2.632 (a)	32.868 ± 3.075 (b)	36.313 ± 2.395 (c)
H_2_O_2_ (μmol mg^-1^FW)		0.186 ± 0.016 (a)	0.275 ± 0.014 (b)	0.320 ± 0.006 (c)
MDA (μmol g^-1^ FW)		3.047 ± 0.235 (a)	4.771 ± 0.207 (b)	5.054 ± 0.334 (c)

DW, dry weight; FW, fresh weight.

The values represented the mean ± SD of three biological replicates. The different letters (a, b, and/or c) indicate significant differences among non-stressed, drought-stressed, and salt-stressed *B*. *napus* plants based on one-way ANOVA (*p* < 0.05).

Previous studies have demonstrated that proline, sucrose, and total soluble sugars are compatible osmolytes for the maintenance of turgor and for the stability of cellular structures under adverse environmental conditions [22]. We further determined the osmolyte levels of *B*. *napus* leaves after 4 h of stress treatments. As shown in Table 1, the proline levels under salt stress and drought stress were 42.93% and 43.85% higher than that observed under control conditions, respectively, both significant differences, whereas the differences in proline content between drought and salt stress were not significant. The total soluble sugar concentration in leaves was elevated by 18.42% under salt stress and by 31.36% under drought stress, and the sucrose concentration in leaves increased by 45.14% under salt stress and by 60.35% under drought stress. ANOVA indicated that the increases in total soluble sugar and sucrose concentrations under drought stress were significantly higher than those observed after salt stress treatment (Table 1).

Generally, the concentrations of MDA and H_2_O_2_ are major indicators of stress-triggered oxidative damage and ROS accumulation [25]. We measured the concentrations of MDA and H_2_O_2_ in leaves. The concentrations of MDA increased by 56.58% under salt stress and by 65.87% under drought stress, whereas H_2_O_2_ levels increased by 47.85% under salt stress and by 72.04% under drought stress. These results indicated that oxidative damage and ROS accumulation in leaves were more severe under drought stress than under salt stress, consistent with the phenotypic results (Table 1).

**Fig 2:**
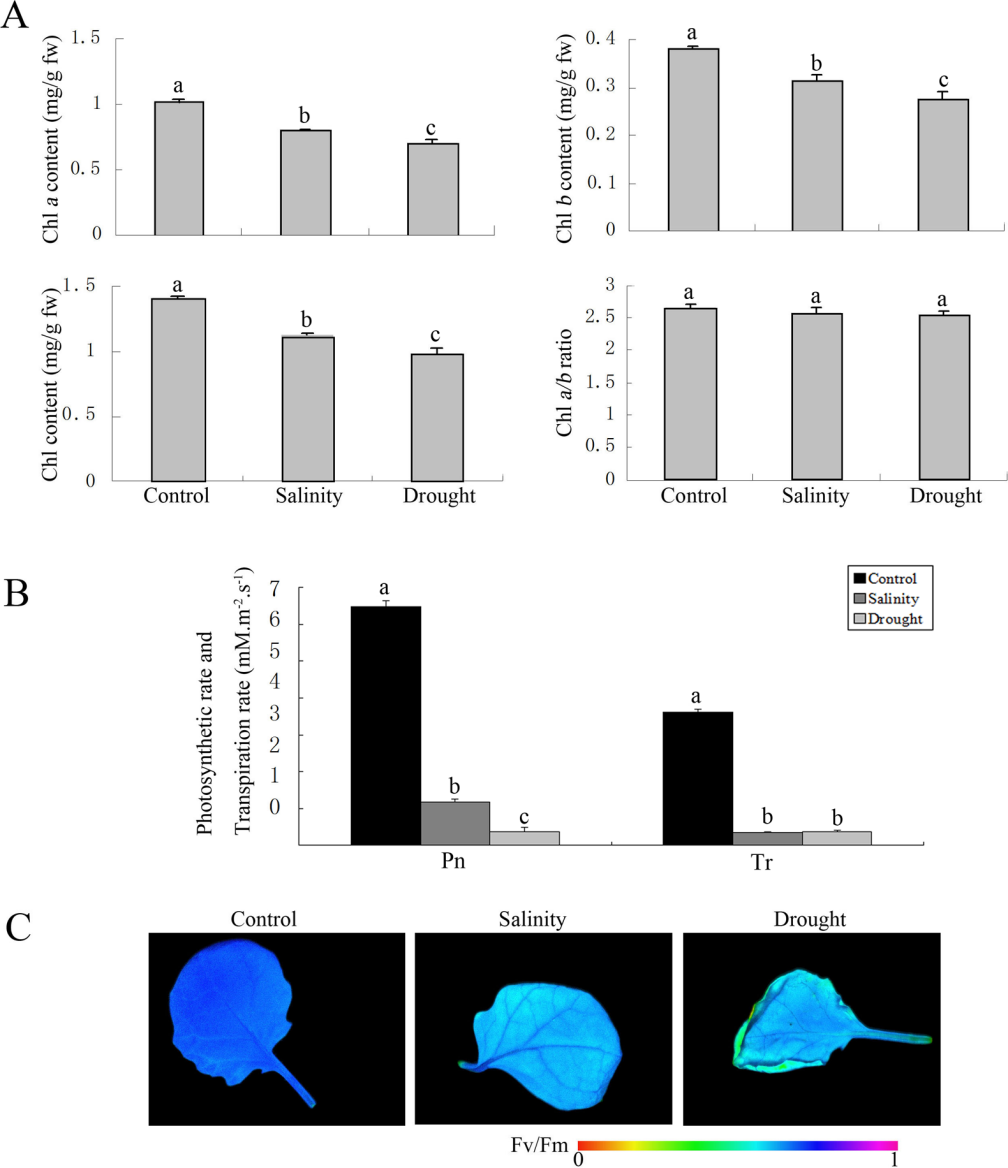
Characterization of salt- and drought-treated *B*. *napus* leaves. (a) Chlorophyll (Chl) *a*, Chl *b*, and total Chl concentration and Chl *a/b* ratio of stress-treated or untreated leaves. Error bars represent SD (*n* = 3). fw, fresh weight. (b) Photosynthetic rate and transpiration rate of stress-treated or untreated leaves. Error bars represent SD (*n* = 3). Pn, Photosynthetic rate; Tr, Transpiration rate. (c) F_v_/F_m_ values of stress-treated or untreated leaves. The color bar indicates F_v_/F_m_ values from 0 to 1. Different letters (a, b, and/or c) indicate significant differences among non-stressed, drought-stressed, and salt-stressed *B*. *napus* plants based on one-way ANOVA (p < 0.05).

To determine whether photosynthetic systems differ in their responses to different abiotic stresses, we measured chlorophyll *a* and *b* concentrations, chlorophyll fluorescence parameters, the net photosynthesis rate and the transpiration rate. Under salt stress, the chlorophyll *a* and *b* contents in *B*. *napus* leaves decreased by 20.9% and 18.1%, respectively, whereas under drought stress, the chlorophyll *a* and *b* content decreased by 30.8% and 20.9%, respectively (Fig 2A). The photosynthetic rate was inhibited by 81.8% under salt stress and by 94.3% under drought stress. The transpiration rate decreased by 90.3% under salt stress and by 90.1% under drought stress (Fig 2B). salt and drought stress-treated leaves exhibited lower F_v_/F_m_ values than did the control (Fig 2C). The adverse effects on photosynthetic systems were more severe under drought stress than under salt stress.

In summary, these results indicated that the physiological responses (e.g., water loss, osmolytes accumulation, oxidative damage, and photosynthesis) which usually were observed during the late stages of stress responses reported previously also occurred in the early stage of stress responses in the present study, and the physiological response under drought stress was stronger than that under salt stress.

### Proteome profile of *B*. *napus* leaves under salt and drought stresses

To further explore the early responses to salt and drought stresses in *B*. *napus*, we analyzed the proteome profiles of *B*. *napus* leaves under salt and drought stresses by iTRAQ-LC/MS-MS. In total, 339,224 spectra were obtained, of which 63,645 spectra were matched to known peptides and 37,623 spectra were categorized as unique peptides. A total of 5,583 unique proteins were identified (S2 and S3 Tables). Among these proteins, 48.5% (2708/5583) had at least two unique peptides, 75% of the peptides length were 8–15 amino acids long, 60% of the proteins had molecular weights of 20–70 kD, and 71% of the proteins exhibited greater than 5% sequence coverage (S1 Fig). These results indicated that the data qualified for further analysis.

**Fig 3:**
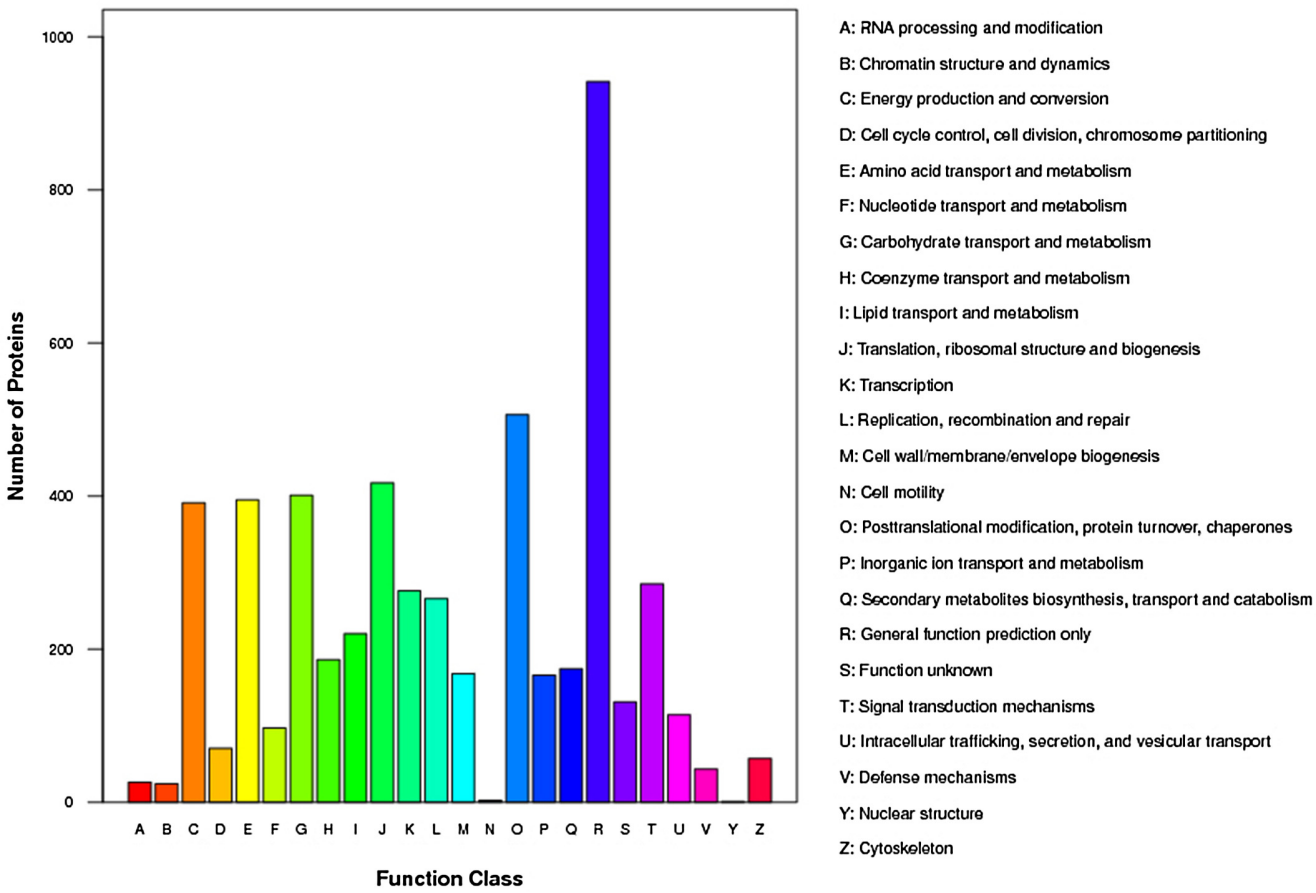
Clusters of Orthologous Groups of proteins (COG) classification of total proteins in *B*. *napus* leaves. A: RNA processing and modification; B: chromatin structure and dynamics; C: energy production and conversion; D: cell cycle control, cell division, chromosome partitioning; E: amino acid transport and metabolism; F: nucleotide transport and metabolism; G: carbohydrate transport and metabolism; H: coenzyme transport and metabolism; I: lipid transport and metabolism; J: translation, ribosomal structure and biogenesis; K: transcription; L: replication, recombination and repair; M: cell wall/membrane/envelope biogenesis; N: cell motility; O: posttranslational modification, protein turnover, chaperones; P: inorganic ion transport and metabolism; Q: secondary metabolites biosynthesis, transport and catabolism; R: general function prediction only; S: function unknown; T: signal transduction mechanisms; U: intracellular trafficking, secretion, and vesicular transport; V: defense mechanisms; Y: nuclear structure; Z: cytoskeleton.

Further functional classification [26] revealed that the proteins were primarily involved in posttranslational modification, protein turnover, chaperon functions, energy production and conversion, amino acid and carbohydrate transport and metabolism, translation, posttranslational modification/protein turnover, and signal transduction (Fig 3). Our results indicated that the proteins expressed in the early responses were involved in nearly every aspect of plant growth and metabolism.

**Fig 4:**
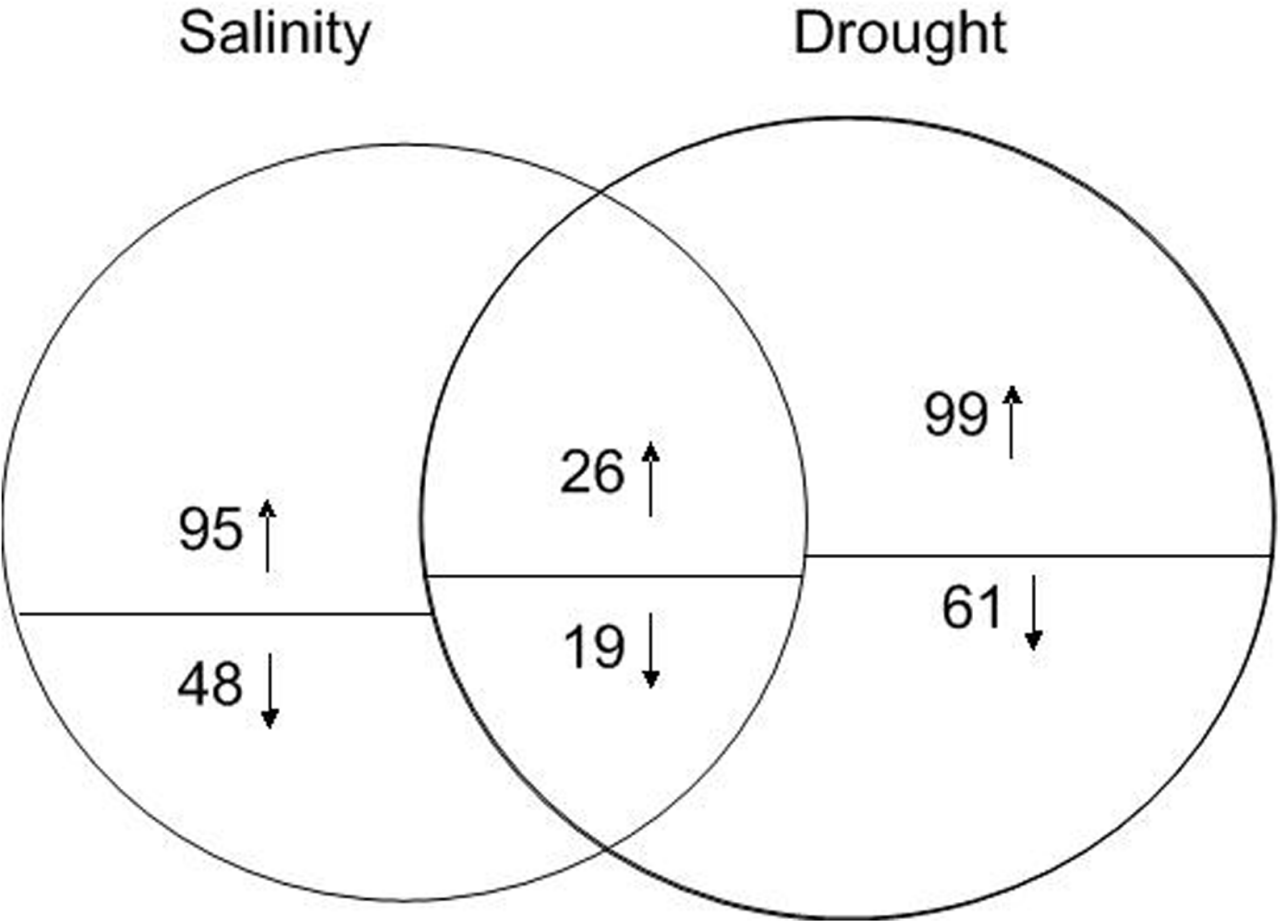
Venn diagram of the distribution of differentially expressed proteins responsive to salt and drought stresses. The circles are proportional to the number of proteins identified in each treatment. The overlapping regions indicate the number of common proteins. The number above or below the horizontal line in each portion indicates the number of up-regulated or down-regulated proteins. In total, 188 and 205 proteins were identified by iTRAQ as salt-responsive and drought-responsive, respectively. Forty-five proteins were identified under both salt and drought treatments. Among the 143 salt-specific DEPs, 95 were up-regulated and 48 were down-regulated. Among 160 drought-specific DEPs, 99 were up-regulated and 61 were down-regulated. Among the 45 common DEPs, 26 were up-regulated and 19 were down-regulated.

To explore the similarities and differences in the proteome profiles of the salt and drought stress responses in *B*. *napus* leaves, we compared the proteome profiles of *B*. *napus* leaves from salt- and drought-treated seedlings with those of the non-treated control. As shown in Fig 4, of the 5,583 unique proteins detected in *B*. *napus* leaves, 188 were differentially expressed between the control and salt-treated samples, and 205 were differentially expressed between the control and drought-treated samples. More DEPs were identified in drought-stressed leaves than in salt-stressed leaves.

### Validation of iTRAQ data for selected candidate proteins by MRM

To study the early responses of *B*. *napus* to salt and drought stresses, the treatment duration should be very short; however, the range of change in the protein profile may be not very obvious within such a short period. To retain as much valuable information as possible, we set comparatively less stringent criteria (see Materials and Methods) to define DEPs in the proteome analysis. Therefore, evaluating the reliability of the data was of great concern.

**Fig 5:**
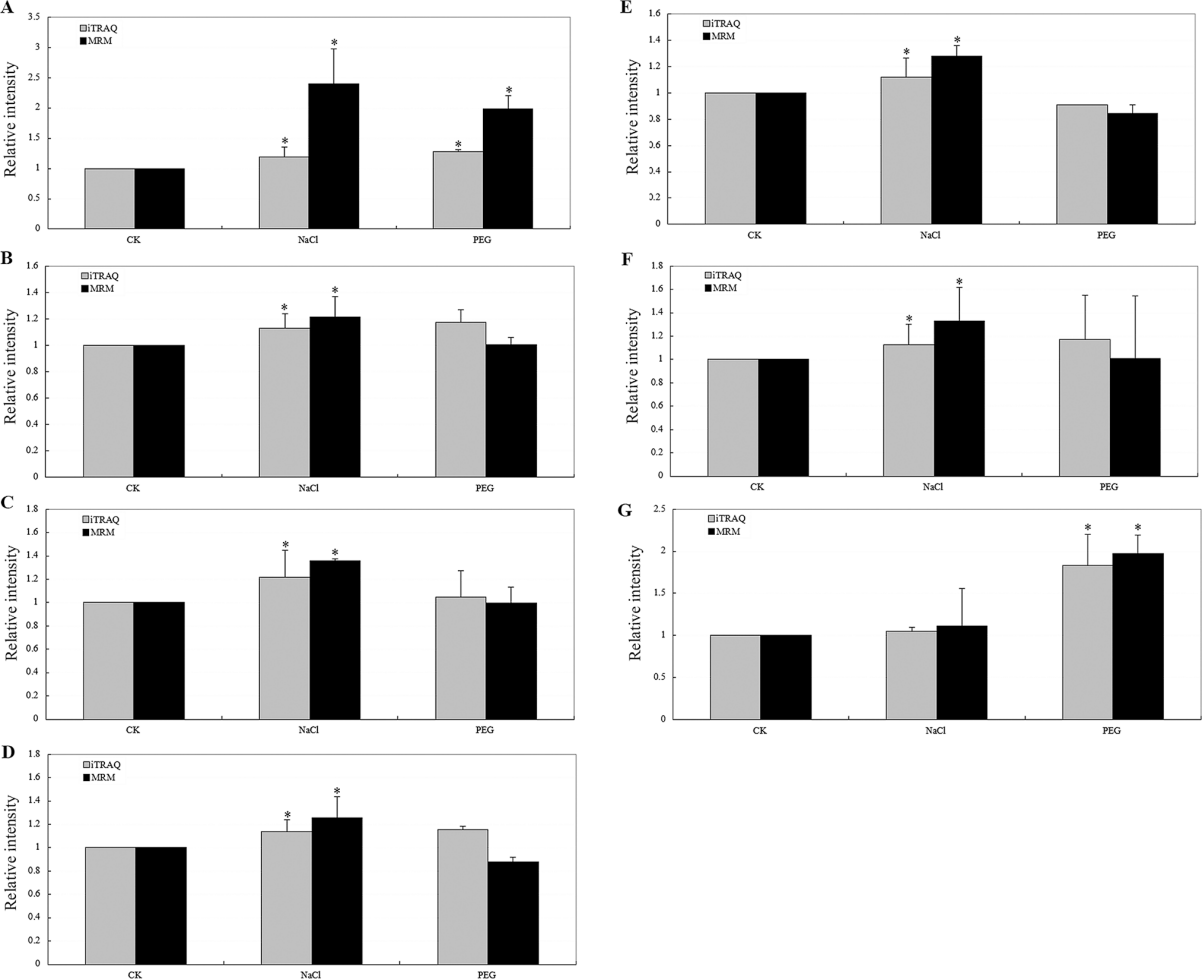
Relative expression levels of selected proteins measured by iTRAQ and MRM in *B*. *napus* leaves under the control, 245 mM NaCl and 25% PEG 6000 treatments for 4 h. (a) FAD-binding and BBE domain-containing protein, (b) chlorophyll *a-b* binding protein CP29.3, (c) TIC110, (d) ATPase 2, (e) thylakoid lumenal 16.5 kDa protein, (f) GPI-anchored protein, (g): carbonic anhydrase. Error bars represent SD (*n* = 3). Asterisks indicate a significant difference compared with the control (*p* < 0.05).

MRM is a fast and highly precise method for quantifying targeted proteins through measurements of representative peptides via mass spectrometry [27], particularly when antibodies directed against the proteins of interest are unavailable. To verify the quantitative data derived from iTRAQ, seven DEPs were randomly selected for validation by MRM. The temporal expression levels of the proteins detected by MRM corroborated well with the iTRAQ data (Fig 5). These results demonstrated that the iTRAQ data were highly reliable for further analysis and that our definition of DEPs was likely adequate to identify real differences between the stress treatments and control.

### Overlapping changes in protein profiles in response to drought and salt stresses

Our previous physiological data indicated that common osmotic stress and the associated oxidative stress may lead to similar physiological and molecular responses to salt and drought stresses. To further determine whether similar trends exist in the proteomic profiles of the early-stage responses to salt and drought stresses, we analyzed the DEPs shared by the two stress treatments. In total, 24% (45/188) and 22% (45/205) of the DEPs were shared by plants under both salt and drought stress treatments, respectively. Excluding two proteins that exhibited opposite trends, 43 common proteins displayed extremely similar change trends in response to the stress treatments (Table 2 and S4 Table).

**Table 2:** Differentially expressed proteins common to the salt and drought stresses responses.

Accession^a^	Protein name	Species	Uniquepeptide	Fold change of NaCl/CK	Fold change of PEG/CK
sp|P14279	Chlorophyll a-b binding protein 5	*Solanum lycopersicum*	3	1.5	1.36
gi|62321409	Carbonic anhydrase	*Arabidopsis thaliana*	2	1.19	1.82
gi|62319619	Xylose isomerase	*Arabidopsis thaliana*	10	1.29	1.2
sp|Q9SVG4	Reticuline oxidase-like protein	*Arabidopsis thaliana*	7	1.2	1.28
gi|9279589	Dihydrolipoamide S-acetyltransferase	*Arabidopsis thaliana*	3	0.83	0.86
sp|Q2V2S7	NAD(P)H-quinone oxidoreductase subunit M	*Arabidopsis thaliana*	3	0.84	0.8
gi|119389583	Allene oxide cyclase 2	*Arabidopsis thaliana*	2	1.62	2.38
sp|Q93XW5	Nitrile-specifier protein 5	*Arabidopsis thaliana*	5	1.6	1.5
sp|Q9SJD4	Long chain acyl-CoA synthetase 8	*Arabidopsis thaliana*	5	1.24	1.35
gi|62319394	Dihydroorotate dehydrogenase like-protein	*Arabidopsis thaliana*	6	1.26	1.29
sp|Q9SXP7	Octanoyltransferase	*Arabidopsis thaliana*	4	1.24	1.21
gi|15229458	Cyanate hydratase	*Arabidopsis thaliana*	3	1.21	0.85
gi|15228596	Cysteine synthase A	*Arabidopsis thaliana*	4	0.81	0.84
gi|15241295	Aspartokinase 2	*Arabidopsis thaliana*	4	0.78	0.81
gi|42572701	Biotin/lipoyl attachment domain-containing protein	*Arabidopsis thaliana*	3	0.71	0.66
gi|6048743	Chitinase	*Brassica juncea*	2	1.49	1.61
gi|347309137	Ascorbate peroxidase	*Brassica oleracea*	6	1.17	1.22
gi|334185637	Peroxiredoxin Q	*Arabidopsis thaliana*	6	0.78	0.77
gi|15229775	40S ribosomal protein S14-2	*Arabidopsis thaliana*	2	1.37	1.25
sp|Q6WZ83	Histone H4	*Eucalyptus globulus*	7	1.41	1.22
sp|Q9SJA6	Serine/arginine-rich splicing factor RSZ22A	*Arabidopsis thaliana*	4	0.78	1.22
sp|Q9SMB8	Tyramine N-feruloyltransferase 4/11	*Nicotiana tabacum*	3	0.9	0.88
sp|P49313	30 kDa ribonucleoprotein	*Nicotiana plumbaginifolia*	3	0.83	0.75
sp|Q6ICX4	Polypyrimidine tract-binding protein homolog 3	*Arabidopsis thaliana*	2	0.76	0.76
gi|20140684	Translationally-controlled tumor protein homolog	*Brassica oleracea*	2	0.88	0.82
gi|118197456	Elongation factor 1-beta	*Brassica rapa*	2	0.88	0.82
gi|119720832	Ribosomal protein S27	*Brassica rapa*	4	0.83	0.86
gi|15239409	60S ribosomal protein L12-3	*Arabidopsis thaliana*	3	0.81	0.84
gi|42564228	MAR-binding filament-like protein 1	*Arabidopsis thaliana*	2	0.73	0.72
gi|62321351	Leucine aminopeptidase	*Arabidopsis thaliana*	3	1.31	1.25
gi|15242045	Chaperonin 20	*Arabidopsis thaliana*	4	0.83	0.8
sp|O22870	Peptidyl-prolyl cis-trans isomerase FKBP16-3	*Arabidopsis thaliana*	4	0.78	0.81
gi|297848776	Calcium-binding EF-hand family protein	*Arabidopsis lyrata*	10	1.33	1.36
gi|18405391	ZKT protein containing PDZ, K-box and a TPR region	*Arabidopsis thaliana*	11	0.83	0.82
gi|110739803	Syntaxin related proteint Vam3p	*Arabidopsis thaliana*	2	1.74	1.56
gi|62320206	Ca^2+^-dependent membrane-binding protein annexin	*Arabidopsis thaliana*	5	1.62	1.25
gi|297843340	Synaptotagmin	*Arabidopsis lyrata*	4	1.43	1.39
sp|Q0WNJ6	Clathrin heavy chain 1	*Arabidopsis thaliana*	2	1.2	1.24
gi|121489751	ABC transporter precursor	*Phillyrea latifolia*	6	1.17	1.23
sp|Q8LC95	Early nodulin-like protein 3	*Arabidopsis thaliana*	3	1.52	1.64
sp|Q9LQU4	PLANT CADMIUM RESISTANCE 2	*Arabidopsis thaliana*	3	1.31	1.53
gi|159138535	Leucine zipper protein	*Helianthus annuus*	2	1.19	1.26
gi|147816472	Hypothetical protein	*Vitis vinifera*	7	1.26	1.3
gi|297800834	Hypothetical protein	*Arabidopsis lyrata*	2	1.12	1.24
gi|312281509	Unnamed protein	*Thellungiella halophila*	3	0.69	0.64

^a^ Accession numbers according to NCBInr (gi) or Swiss-Prot (sp).

The functions of those proteins were categorized according to biological functions that included photosynthesis, carbohydrate and energy metabolism, metabolism, stress and defense, transcription- and translation-related, protein folding and degradation, signaling, membrane and transport (S4 Table). Approximately 24% (11/45) of the proteins were transcription- and translation-related, indicating that both stresses clearly affected gene transcription and protein translation. Approximately 73% of those proteins (8/11) were down-regulated in both stress treatments, i.e., 60S ribosomal protein L12-3, ribosomal protein S27, 30 kDa ribonucleoprotein, and elongation factor 1-beta. Furthermore, all proteins in the category of membrane and transport were up-regulated. These proteins included vam3p, annexin, synaptotagmin, clathrin heavy chain 1, and ABC transporter precursor, showing that up-regulation of these proteins may re-establish the cellular osmotic balance and *in vivo* compartmentalization under both stress treatments, consistent with a previous report [26].

### Profiles of drought- and salt-specific differentially expressed proteins

**Fig 6:**
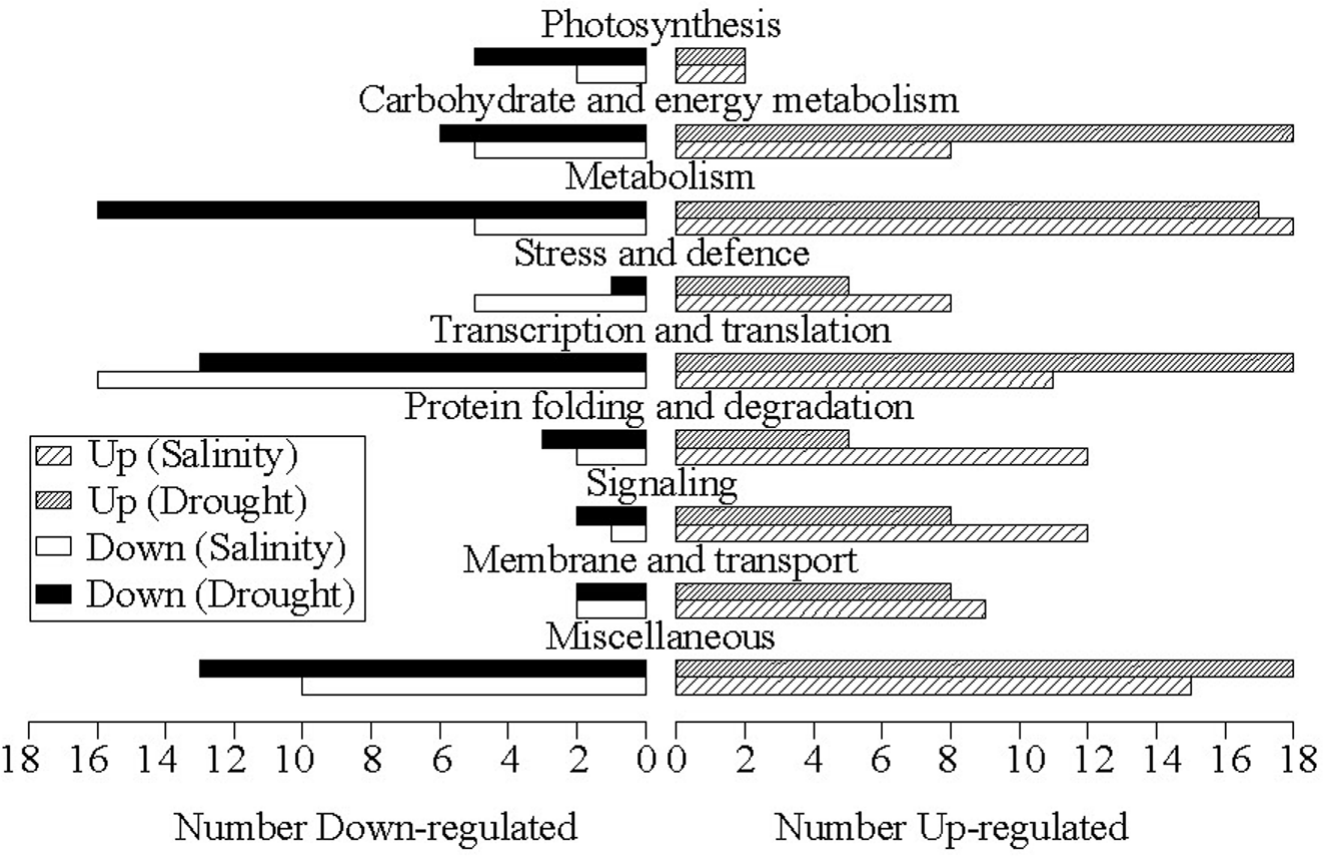
Expression patterns of salt stress-specific and drought stress-specific differentially expressed proteins in each functional category.

Based on pairwise comparisons, 143 salt stress-specific DEPs, including 95 up-regulated and 48 down-regulated proteins, and 160 drought stress-specific DEPs, including 99 up-regulated and 61 down-regulated proteins, were identified (Fig 4). Further characterization of the functional features of the DEPs revealed that they were involved in a wide range of biological processes, including photosynthesis, carbohydrate and energy metabolism, metabolism, stress and defense, transcription- and translation-related, protein folding and degradation, signaling, membrane and transport, and “miscellaneous” (Fig 6, S5 and S6 Tables).

Fig 6 presents the overall response pattern of *B*. *napus* leaves to specific salt or specific drought stress based on their proteomic profiles. Two common features were observed by pairwise comparison of both stress responses: 1) overall, more proteins were up-regulated than down-regulated, regardless of the actual stressor; 2) two key categories associated with growth, “metabolism” and “transcription and translation”, notably changed under both salt and drought stresses. Several differences in stress responses were also observed: 1) there were more drought-specific responsive DEPs than salt-specific responsive DEPs, and more proteins were down-regulated proteins in response to drought than to salt stress; 2) more photosynthesis- and metabolism-related proteins were down-regulated in response to drought, whereas more protein folding- and degradation-associated proteins and signaling proteins were up-regulated in response to salt. These proteomic features, including greater activation of transcription and translation, more severe attenuation of metabolism, and greater inhibition of photosynthesis-related proteins in response to drought, were consistent with the more severe physiological responses to drought stress in *B*. *napus*. Meanwhile, the proteomic changes revealed greater number of active protein folding- and degradation-associated proteins and signaling proteins in response to salt, consistent with the physiological phenomenon of enhanced salt tolerance in *B*. *napus* (Figs 1 and 2, Table 1).

To further understand the changes in the proteome profiles between the salt and drought stress responses, pathway enrichment analysis was performed. Compared to the control, proteins involved in signaling, membrane and transport and photosynthesis were largely enriched in both salt and drought stress responses. Therefore, these proteins were selected for further analysis.

### DEPs related to signal perception and transduction

Signaling components play essential roles in plant adaptions to abiotic stresses. In total, 25 signaling-related proteins were identified, including two overlapping DEPs in both treatments, 13 salt-specific DEPs and 10 drought-specific DEPs. The majority of these proteins were up-regulated (Table 3). Based on the roles of these proteins in signal perception and transduction pathways, we classified these proteins into several groups, including probable receptors, small GTP-binding proteins, and Ca^2+^ sensors (Table 3).

**Table 3:** Signaling-related proteins differentially expressed under salt and drought stresses.

	NaCl/CK				PEG/CK			
	Accession^a^	Protein name	Fold change	Species	Accession^a^	Protein name	Fold change	Species
(1) Probable receptor	gi|110736873	GPI-anchored protein	1.13	*Arabidopsis thaliana*	sp|Q9SYM9	Receptor-like serine/threonine-protein kinase	1.17	*Arabidopsis thaliana*
	sp|Q8L710	Cysteine-rich receptor-like protein kinase 17	1.17	*Arabidopsis thaliana*	sp|Q8GYA4	Cysteine-rich receptor-like protein kinase 10	1.28	*Arabidopsis thaliana*
	sp|Q8VZG8	Probable LRR receptor-like serine/threonine-protein kinase	1.65	*Arabidopsis thaliana*	sp|Q9M8T0	Probable inactive receptor kinase	1.21	*Arabidopsis thaliana*
					sp|O48788	Probable inactive receptor kinase	1.45	*Arabidopsis thaliana*
					sp|Q9C9Y8	Probable inactive receptor kinase	1.19	*Arabidopsis thaliana*
(2) Samll G protein related	sp|P31582	Ras-related protein RABF2a	1.23	*Arabidopsis thaliana*	gi|357470519	Ran-binding protein	0.83	*Medicago truncatula*
	sp|P92963	Ras-related protein RABB1c	1.21	*Arabidopsis thaliana*				
(3) Phospholipase	sp|Q39033	Phosphoinositide specific phospholipase C	1.22	*Arabidopsis thaliana*				
(4) Ca^2+^ sensor	gi|297848776	Calcium-binding EF-hand family protein	1.33	*Arabidopsis lyrata*	gi|297848776	Calcium-binding EF-hand family protein	1.36	*Arabidopsis lyrata*
(5) 14-3-3	sp|P48348	14-3-3-like protein GRF8	1.19	*Arabidopsis thaliana*	sp|Q01525	14-3-3-like protein GRF2	1.19	*Arabidopsis thaliana*
(6) Kinases/phosphatases	sp|Q9ZSA2	Calcium-dependent protein kinase 21	1.56	*Arabidopsis thaliana*	gi|56693619	Protein kinase C conserved region 2	1.23	*Brassica napus*
	sp|Q9FHD7	Probable serine/threonine-protein kinase	1.39	*Arabidopsis thaliana*	sp|Q9S713	Serine/threonine-protein kinase STN7	1.17	*Arabidopsis thaliana*
	sp|Q9FR53	Serine/threonine-protein kinase TOR	1.28	*Arabidopsis thaliana*	sp|Q8L7U5	Serine/threonine-protein phosphatase BSL1	0.86	*Arabidopsis thaliana*
	sp|Q05609	Serine/threonine-protein kinase CTR1	1.25	*Arabidopsis thaliana*				
	sp|Q9SL76	Putative protein phosphatase 2C	1.32	*Arabidopsis thaliana*				
	sp|Q94AT1	Probable protein phosphatase 2C	0.85	*Arabidopsis thaliana*				
(7) Other	gi|18405391	ZKT	0.83	*Arabidopsis thaliana*	gi|18405391	ZKT	0.82	*Arabidopsis thaliana*

^a^ Accession numbers according to NCBInr (gi) or Swiss-Prot (sp).

Two common signaling-related DEPs that responded to both salt and drought stresses were identified: a calcium-binding EF-hand family protein and a novel probable ZKT protein (Table 3). Three potential salt-specific response receptors and five potential drought-specific response receptors were also identified. Receptor-like kinases (RLKs) belong to a kinase superfamily that regulates plant growth and behavior [28]. In the salt stress response, two RLKs consisting of a cysteine-rich receptor-like protein kinase 17 (CRK17) and a probable LRR receptor-like serine/threonine-protein kinase (LRR-RLK) were identified. Two RLKs, a cysteine-rich receptor-like protein kinase 10 (CRK10) and receptor-like serine/threonine-protein kinase were identified in the drought stress response. Interestingly, we identified a probable receptor protein in the salt stress response, glycosylphosphatidylinositol (GPI)-anchored protein, which has not been reported to be involved in salt or drought stress responses. These receptors were all up-regulated. Moreover, during downstream signal transduction, we identified three small G proteins. RABF2a and RABB1c were up-regulated by 23% and 21% under salt stress, whereas a Ran-binding protein exhibited a 17% reduction under drought stress. Furthermore, we found that the 14-3-3 protein GF14 kappa (GRF8) was up-regulated under salt stress, whereas the 14-3-3-like protein GF14 omega (GRF2) was up-regulated under drought stress. Three serine/threonine-protein kinases, one calcium-dependent protein kinase 21 (CDPK 21), and one putative protein phosphatase 2C displayed significant accumulation in response to salt stress. Another probable protein phosphatase 2C exhibited decreased expression during salt stress (Table 3). By contrast, two kinases were up-regulated, and one serine/threonine-protein phosphatase, BSL1, was down-regulated under drought stress (Table 3). In addition, we identified an up-regulated phosphoinositide-specific phospholipase C under salt stress but not under drought stress in the present study. These results indicated that early signal perception and transduction may be enhanced under salt and drought stresses.

### DEPs related to ion transport and vacuolar trafficking

ABC transporters function in the transport of hormones, lipids and secondary metabolites, which are widely reported to be associated with abiotic stress responses [26, 29]. The present proteome analysis indicated that the ABC transporter precursor was up-regulated in both salt and drought stress treatments (Table 4). The subfamilies ABC transporter B and ABC transporter C were specifically detected under salt stress, whereas the subfamily ABC transporter G was specifically identified under drought stress. These differences in expression indicated that different gene family members executed different functions to cope with various stresses. All ABC transporters were up-regulated under both stress treatments (Table 4). Different TIC family proteins that play roles in chloroplast protein import trafficking were identified (Table 4). In addition, two plasma-membrane-localized H^+^-ATPases were up-regulated in *B*. *napus* leaves under salt stress; however, a vacuole-localized H^+^-ATPase was down-regulated under drought stress. A Ca^2+^-dependent membrane-binding protein, annexin D1 (ANN1), was up-regulated under both salt and drought stresses in *B*. *napus* leaves.

**Table 4:** Membrane and transport proteins differentially expressed under salt and drought stresses.

	NaCl/CK				PEG/CK			
	Accession^a^	Protein name	Fold change	Species	Accession^a^	Protein name	Fold change	Species
(1) ABC transporter	gi|121489751	ABC transporter precursor	1.17	*Phillyrea latifolia*	gi|121489751	ABC transporter precursor	1.23	*Phillyrea latifolia*
	sp|Q8LPQ6	ABC transporter B family member 28	1.15	*Arabidopsis thaliana*	sp|Q93YS4	ABC transporter G family member 22	1.23	*Arabidopsis thaliana*
	sp|Q9LZJ5	ABC transporter C family member 14	1.25	*Arabidopsis thaliana*	sp|Q9XIE2	ABC transporter G family member 36	1.15	*Arabidopsis thaliana*
(2) Other transporter	sp|Q9ZPY7	Exportin-2	1.35	*Arabidopsis thaliana*	gi|13877587	2-Oxoglutarate/malate translocator proteinprecursor-like protein	1.22	*Arabidopsis thaliana*
	sp|Q8LPR9	TIC110	1.22	*Arabidopsis thaliana*	gi|28540971	TPT	1.34	*Brassica rapa*
	sp|Q9FMD5	TIC 40	0.89	*Arabidopsis thaliana*	gi|30694885	TPT	1.52	*Arabidopsis thaliana*
	sp|Q9FJX3	Mitochondrial outer membrane protein porin 2	1.20	*Arabidopsis thaliana*				
	sp|Q93XM7	Mitochondrial carnitine/acylcarnitine carrier-like protein	0.83	*Arabidopsis thaliana*				
(3) ATPase	sp|P20431	H+-ATPase 3, plasma membrane-type	1.38	*Arabidopsis thaliana*	sp|Q8W4E2	V-type proton ATPase subunit B3	0.83	*Arabidopsis thaliana*
	sp|P19456	ATPase 2, plasma membrane-type	1.14	*Arabidopsis thaliana*	sp|Q9XES1	Calcium-transporting ATPase 4	1.32	*Arabidopsis thaliana*
(4) Ion channel	sp|Q9SYT0	Ca^2+^-dependent membrane-binding protein annexin D1	1.62	*Arabidopsis thaliana*	sp|Q9SYT0	Ca^2+^-dependent membrane-binding protein annexin D1	1.25	*Arabidopsis thaliana*
(5) Vesicle trafficking	gi|110739803	Syntaxin related protein AtVam3p	1.74	*Arabidopsis thaliana*	gi|110739803	Syntaxin related protein AtVam3p	1.56	*Arabidopsis thaliana*
	sp|Q0WNJ6	Clathrin heavy chain 1	1.20	*Arabidopsis thaliana*	sp|Q0WNJ6	Clathrin heavy chain 1	1.24	*Arabidopsis thaliana*
	gi|297843340	Synaptotagmin	1.43	*Arabidopsis lyrata*	gi|297843340	Synaptotagmin	1.39	*Arabidopsis lyrata*
	sp|Q5U247	Exocyst complex component 8	1.33	*Xenopus laevis*	sp|Q8L828	Coatomer subunit beta'-3	1.23	*Arabidopsis thaliana*
	sp|P31582	Ras-related protein RABF2a	1.23	*Arabidopsis thaliana*	sp|Q9C827	Coatomer subunit beta'-2	1.53	*Arabidopsis thaliana*
	sp|P92963	Ras-related protein RABB1c	1.21	*Arabidopsis thaliana*	sp|Q8LPK4	AP-2 complex subunit alpha-2	0.86	*Arabidopsis thaliana*
					gi|357470519	Ran-binding protein	0.83	*Medicago truncatula*

^a^ Accession numbers according to NCBInr (gi) or Swiss-Prot (sp).

Furthermore, we identified 11 vesicle trafficking-related proteins that exhibited differential expression patterns under salt and drought stresses (Table 4). Among these proteins, four proteins overlapped in both the salt and drought stress responses, including clathrin heavy chain 1, syntaxin-related protein AtVam3p, synaptotagmin and Ca^2+^-dependent membrane-binding protein annexin D1. Exocyst complex component 84B was a salt-response-specific DEP. Coatomer subunit beta'-3, coatomer subunit beta'-2 and AP-2 complex subunit alpha-2 were identified as drought response-specific DEPs. In addition, three proteins belonging to small G protein family proteins (RABF2a, RABB1c and a Ran-binding protein) that function not only in single transduction but also in membrane trafficking were identified. Thus, the exchange of products between different endomembrane systems was adjusted under salt and drought stresses, and the adjustment modes differed between salt and drought stresses.

### DEPs related to photosynthesis

Previous study have showed that salt or drought stress can affect the photosynthetic rate and enzyme activities associated with CO_2_ assimilation [30]. In the present proteomic analysis, we also identified a set of DEPs associated with the photosynthetic system in *B*. *napus* leaves (S4–S6 Tables). These DEPs exhibited diverse responses to the stress treatments. i) Two proteins, chlorophyll *a/b* binding protein 5 and carbonic anhydrase, which functions in light harvesting and the Calvin cycle, were up-regulated in both stress treatments. ii) Four DEPs were salt response specific: two down-regulated chlorophyll *a-b* binding proteins, CP26 and Lhca2, the up-regulated chlorophyll *a-b* binding protein CP29.3, and an up-regulated thylakoid lumenal 16.5 kDa protein. iii) Seven DEPs were drought response specific, including two up-regulated and five down-regulated proteins. Notably, THYLAKOID FORMATION 1 (THF1), which is required for dynamic of PSII-LHCII supramolecular organization in higher plants by interacting with Lhcb, was up-regulated [31]. Three of the five down-regulated proteins were light reaction process-related proteins: oxygen-evolving enhancer protein 2, photosystem I reaction center subunit III, and PsbP domain-containing protein 4. The other two down-regulated proteins were ferredoxin or ferredoxin-like proteins. Ferredoxin, an electron carrier in the photosynthetic electron transport chain, accepts electrons from photosystem I and delivers them to essential oxido-reductive pathways. Taken together, most of these DEPs were up-regulated under salt stress treatment and down-regulated under drought stress. These results suggest that *B*. *napus* implements divergent mechanisms to modulate the photosynthetic machinery in response to different stresses.

## Discussion

### Systematic differences and similarities of the responses to salt and drought stresses

The physiological data indicated that most of the stress responses were similar between the salt and drought treatments, such as a decreased water content, accumulation of osmoprotectants such as proline and sugar, increased oxidative damage, and attenuation of the photosynthetic rate. However, the drought response was more severe than the salt response, revealing the major differences between salt and drought stresses. Excluding the potential effect of PEG, the only reason to explain this phenomenon is that *B*. *napus* is a relatively salt-tolerant species [2]. This result also indicated that the salt stress response in *B*. *napus* is not a simple superposition of water stress and ion toxicity.

Comparison of proteome profile changes between the salt and the drought responses revealed that DEPs associated with the photosynthetic system were significantly enriched. A greater number of proteins involved in the light reaction were down-regulated in the drought stress treatment compared to that of salt stress treatment, consistent with the lower photosynthesis rate observed under drought stress. Huang et al. reported that chlorophyll content decreases more slowly in the *thf1* mutant of *Arabidopsis*. In the present study, THF1 was specifically up-regulated under drought stress. Therefore, we speculated that, the decrease in chlorophyll *a* and *b* concentrations was greater under drought stress than under salt stress, which may be a result of up-regulation of THF1 under drought stress. Moreover, the photosynthetic rate fell by 94.3% under drought stress, significantly higher than the 81.8% decrease observed under salt stress (Fig 2). Thus, the accumulation of organic compounds and energy supply in *B*. *napus* was lower for 4 h of drought stress treatment compared to 4 h of salt treatment, consistent with the greater number of down-regulated proteins in the “metabolism” category in the drought stress response in our proteome data.

**Fig 7:**
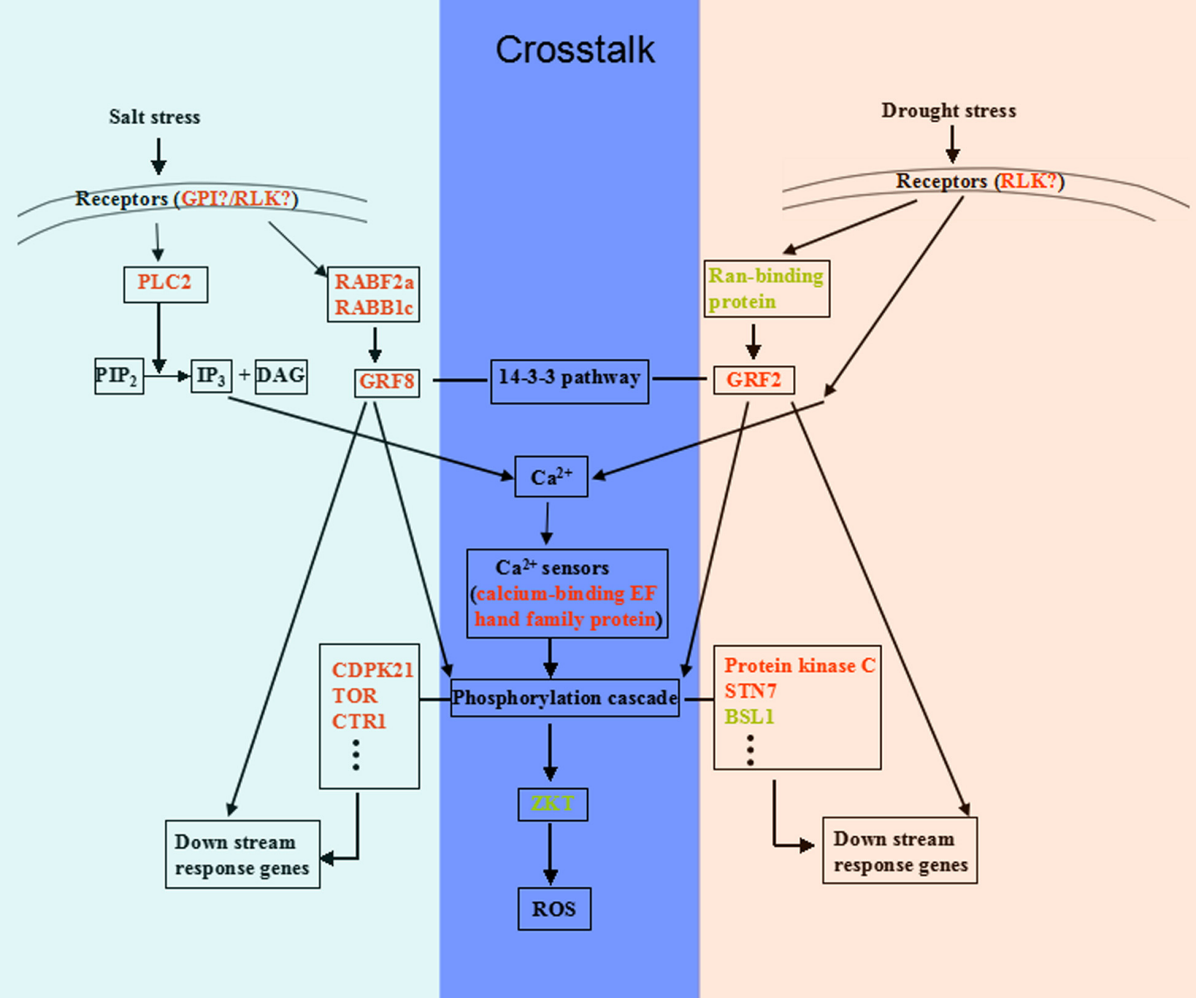
Cross-talk and specificity of signal perception and transduction in response to salt and drought stress in *B*. *napus*. The most differentially expressed signaling-related proteins were integrated and marked in red (up-regulated) or green (down-regulated). The blue region in the middle represented the cross-talk pathway shared by both the salt and drought responses. The calcium-bind EF-hand family protein, which acted as a Ca^2+^ sensor sensing Ca^2+^ ion concentration in the cytoplasm, and ZKT protein, which may act as a molecular adaptor that was regulated by phosphorylation and modulated the ROS pathway, were overlapping signaling proteins in the salt and drought responses. Differential signal receptors, small G protein, 14-3-3 pathway members, kinases and phosphatases were identified in the salt and drought responses, indicating differences in signal perception and transduction occurred in the early stage of the salt and drought responses. Abbreviations: GPI, glycosylphosphatidylinositol (GPI)-anchored protein; PLC2, phospholipase C; RABF2a, Ras-related protein RABF2a; RABB1c, Ras-related protein RABB1c; GRF8, 14-3-3-like protein GF14 kappa; GRF2, 14-3-3-like protein GF14 omega; CDPK21, calcium-dependent protein kinase 21; TOR, serine/threonine-protein kinase TOR; CTR, serine/threonine-protein kinase CTR, STN7, serine/threonine-protein kinase STN7; BSL1, serine/threonine-protein phosphatase BSL1; ZKT, protein containing PDZ, K-box and a TPR region.

The proteome data did not provide direct evidence to support physiological data such as the higher accumulation of sugar and sucrose or more serious of oxidative damage in the drought response. However, it is well known that these types of down-stream responses usually lag early signal perception and transduction. By analyzing of the details of the potential functions of DEPs in signaling, we summarized the putative signal regulation networks in salt and drought stress responses (see Fig 7 for the pathways). We speculated that the cross-talk and divergence of signaling pathways just underlie the similarities and differences in the proteome profiles and physiological responses under different stress treatments.

Signal perception is the first step for plant adaptation to abiotic stresses. Adverse stimuli usually are perceived by diverse receptors anchored on the plasma membrane. However, direct salt sensors or drought sensors have not been identified. Our proteome analysis identified an interesting protein, GPI-anchored protein, which has diverse functions and is important for signal transduction, cell-cell interaction, cell adhesion, and host defense [32–34]. In mammalian and yeast cells, some GPI-anchored proteins function as receptors for viruses and toxins [34]. Association with specialized lipids, called a lipid microdomain or lipid raft, is one of the most striking features of GPI-anchored proteins. This lipid microdomain may facilitate the perception of an extracellular or intercellular signal and is required for the selective transport of proteins [35–37]. Downstream signal transduction is mediated by the specific cleavage of GPI-anchored proteins to release GPI and form multiple signal materials as second messengers involving the mobilization of Ca^2+^, activation of DNA synthesis and rebuilding of cell structure. The specific cleaved enzymes include glycosylphosphatidyl-inositol-specific phospholipidase D (GPI-PLD), glycosylphosphatidyl-inositol-specific phospholipase C (GPI-PLC) and phosphatidyl-inositol-specific phospholipase C (PI-PLC) [33]. Interestingly, we indeed identified an up-regulated phosphoinositide-specific phospholipase C in the salt treatment but not the drought treatment in the present study (Table 3, Fig 7). This finding may indicate a potential novel signal perception and signal transduction pathway that is specific to the salt stress response in plant cells, i.e., salt stress signal →GPI-anchored proteins → PI-PLC → Ca^2+^→ protein phosphorylation cascade (Fig 7). In downstream signal transduction, we identified three small G proteins, RABF2a, RABB1c, and Ran-binding protein. Rab and Ran belong to the small G protein superfamily, which function in cell signaling through the receptor-Ras-14-3-3-MAPK pathway and protein trafficking. In this study, the 14-3-3-like protein GF14 kappa (GRF8)-specific response to salt and the 14-3-3-like protein GF14 omega (GRF2)-specific response to drought were identified. 14-3-3 family proteins are master regulators of signal transduction that bind to phosphorylated proteins and transduce the phosphorylation signals to targets. Their regulating target proteins include kinases (e.g., CDPK and MAPK), transcription factors, structural proteins, ion channels, and other functional proteins [26, 38]. Reversible phosphorylation reactions catalyzed by protein kinases and phosphatases are an important part of signal transduction, which involve various physiological and pathological processes. Four kinases, including CDPK21, TOR, CTR and a Probable serine/threonine-protein kinase and two PP2C phosphatases specifically responded to salt, whereas one protein kinase C, STN7 and one BSL1 phosphatase specifically responded to drought. No common kinases or phosphatases were identified in both stress responses, suggesting that different phosphorylation pathways may utilize different kinases/phosphatases to cope with different stresses (Fig 7). These findings further support the above viewpoint regarding the functional specialization of family proteins when encountering different stresses. In addition, our study indicated that two common signaling-related DEPs, calcium-binding EF-hand family protein and ZKT protein, could be key nodes of cross-talk between salt and drought stresses. Calcium-binding EF hand family protein is a type of Ca^2+^ sensor [39] that can recognize the changes in [Ca^2+^]_cyt_ and regulate the activity of specific protein targets in the cell [40]. The observed increase in the expression level of the calcium sensor indicated that calcium, as a second messenger, may mediate both salt and drought stress signaling pathways in *B*. *napus* leaves (Fig 7). Furthermore, ZKT protein, which contains PDZ, K-box and TPR domains, was down-regulated under both salt and drought stresses. In animals, there is no ZKT homologue, but only the PDZ domain containing proteins involved in regulating functions of G protein-coupled receptors, mediating signal transduction pathways, and assembling a variety of membrane-associated proteins [41]. Previous studies in animals have shown that the proteins rich in the PDZ domain usually act as key nodes in complex protein—protein interaction networks [42, 43]. In plants, the ZKT protein acts as a molecular adaptor that is regulated by phosphorylation in the wound response [44]. Zhu *et al*. (2007) discovered that the protein ENH1, which contains the PDZ and rubredoxin domains, plays a role in the detoxification of ROS under salt stress and may involve SOS2 in this detoxification pathway [45]. In the present study, the PDZ domain-containing protein ZKT in *B*. *napus* exhibited a remarkably consistent change trend under both salt stress and drought stress, suggesting that the PDZ domain may participate in regulating cell signaling (From the phosphorylation cascade → ZKT → ROS) and shedding new light on the functions of proteins containing the PDZ domain in the cross-talk between salt and drought stress signal transduction in plants (Fig 7).

### Characteristics of the early salt and drought stress responses

In the present study, 26 signaling-related and 27 transport- and vesicle trafficking-related proteins were identified as DEPs. Signaling-related proteins generally exhibit rapid turnover and low abundance [46]. Surprisingly, many signaling-related DEPs were identified in leaves in the current study using the traditional iTRAQ approach, but not using a phosphoproteomic method or in specific subcellular organelles (e.g., the plasma membrane). We speculated that the 4-h treatment duration was appropriate for the proteomic analysis to inspect signaling pathways of early stress responses in the present experiment. It is helpful to explain why so many transcription- and translation-related proteins were differentially expressed, whereas the stress-, defense-, and metabolism-related proteins exhibited smaller changes in the levels of expression. These results indicated that stress signal perception and transduction was a key component of the early salt and drought stress responses in *B*. *napus* leaves and that transport and vesicle trafficking may play important roles in this process. These observation were consistent with a previous report regarding the early drought stress response of *Arabidopsis* [16]. cutive function were differentling At the early stress response stage, we observed that the differences in the proteome profiles of *B*. *napus* leaves were greater than the similarities between the salt and drought stress treatments. Only 24% and 22% of the DEPs in the salt- and drought-stress treatments, respectively, were shared, lower than the proportions (50%-70%) reported in previous studies of the late stress response [10, 47]. By comparing the proteome profiles of cross-talk and the specificity of the salt and drought stress signal perception and transduction pathways, we identified that only two common proteins that act as convergence nodes in the salt and drought signaling pathways, a Ca^2+^ sensor and ZKT protein (Fig 7). Although both G protein-related signaling and 14-3-3 signaling pathways were identified in both stress responses, the detail proteins of executive functions were different. Thus, the differences in the proteomic profiles between drought-treated and salt-treated seedlings exceeded the similarities in the early stress response, suggesting that the different mechanisms underlying the salt and drought stress responses differ at the early stage but not at the late stage in plants.

## Conclusion

We used iTRAQ-MS/MS to compare the early protein profiles of responses of *B*. *napus* under salt and drought stresses. We identified important cross-talk and divergence of the early response mechanisms between these two stresses. First, the differences in the proteomic profiles between the drought- and salt-treated seedlings exceeded the similarities in the early stress responses. Second, signaling, transport and membrane trafficking, and photosynthesis pathways were important response events in the mechanism underlying the cross-talk and divergence of the early salt and drought response mechanisms of *B*. *napus*. Third, Ca^2+^, G protein-related, 14-3-3 signaling pathways and phosphorylation cascades were shared signal transduction pathways in the salt and drought stress responses, but the specific proteins of executive function differed. Our results revealed functional specialization of protein families when plant cells encountering different stresses. Fourth, only calcium-binding EF-hand family protein, which mediates the Ca^2+^ signature in the cytoplasm, and ZKT, which regulates the ROS pathway, were definitively identified as cross-talk nodes in the salt and drought signaling pathways.

## Supporting Information

S1 Fig (A) Peptide length distribution, (B) peptide number distribution, (C) protein mass distribution, and (D) distribution of protein’s sequence coverage.
(TIF)

S1 Table Osmotic potential of NaCl solutions and PEG 6000 solutions of different concentrations. (XLS)

S2 Table Overview of protein identification results of *B*. *napus* leaf proteins by the iTRAQ-LC/MSMS method. (XLS)

S3 Table Raw iTRAQ data for protein identification and quantitation. (XLS)

S4 Table Common differentially expressed proteins (DEPs) in the leaves of *B*. *napus* treated with 245 mM NaCl or 25% PEG 6000 for 4 h. (XLS)

S5 Table Salt-specific DEPs in the leaves of *B*. *napus* treated with 245 mM NaCl for 4 h. (XLS)

S6 Table Drought-specific DEPs in the leaves of *B*. *napus* treated with 25% PEG 6000 for 4 h. (XLS)
