# Supplementary figures and images for: Elucidation of Cross-Talk and Specificity of Early Response Mechanisms to Salt and PEG-Simulated Drought Stresses in Brassica napus Using Comparative Proteomic Analysis
Source: PLoS One. 2015 Oct 8;10(10):e0138974. doi: 10.1371/journal.pone.0138974 (PMC4598015; doi:10.1371/journal.pone.0138974)

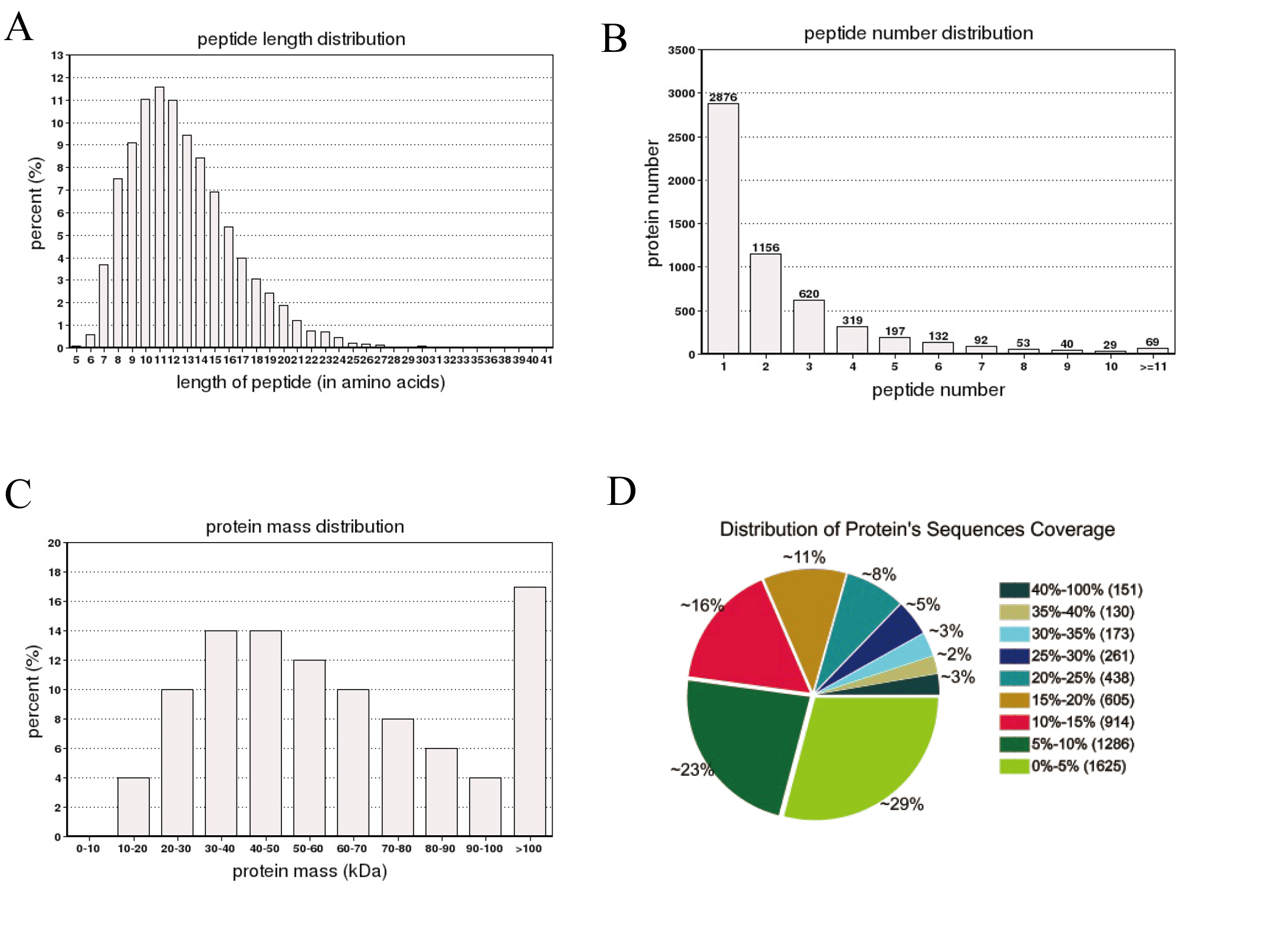

Supplement: S1 Fig — (A) Peptide length distribution, (B) peptide number distribution, (C) protein mass distribution, and (D) distribution of protein’s sequence coverage. (TIF) [file pone.0138974.s001.tif]
